# Green synthesis of *Piper chaudocanum* stem extract mediated silver nanoparticles for colorimetric detection of Hg^2+^ ions and antibacterial activity

**DOI:** 10.1098/rsos.220819

**Published:** 2023-02-08

**Authors:** Khieu Thi Tam, Nguyen Thi Thuy, Nguyen Thi Kim Ngan, Nguyen Manh Khai, Dang Van Thanh

**Affiliations:** ^1^ Faculty of Chemistry, TNU-University of Sciences, Tan Thinh Ward, Thai Nguyen City, Vietnam; ^2^ Department of Environmental Engineering, International University, Quarter 6, Linh Trung Ward, Thu Duc City, Ho Chi Minh City, Vietnam; ^3^ Vietnam National University Ho Chi Minh City, Linh Trung Ward, Thu Duc City, Ho Chi Minh City, Vietnam; ^4^ Faculty of Environmental Sciences, University of Science, Vietnam National University, Hanoi, 334 Nguyen Trai Road, Ha Noi City, Vietnam; ^5^ TNU-University of Medicine and Pharmacy, Thai Nguyen city, Vietnam

**Keywords:** green synthesis, *Piper chaudocanum*, silver nanoparticles, colorimetric detection, Hg^2+^, antibacterial

## Abstract

A green synthetic approach to synthesize silver nanoparticles (AgNPs) using the stem extract of *Piper chaudocanum* for highly sensitive colorimetric detection of Hg^2+^ with a low limit of detection of 23 nM and easy colorimetric read-out has been reported. In addition, the biosynthesized AgNPs demonstrated efficient antibacterial activity against *Escherichia coli*, *Pseudomonas aeruginosa* and *Staphylococcus aureus*. The morphology and structure of the as-synthesized AgNPs were examined using SEM, TEM, EDX, XRD and FT-IR analyses. The XRD and TEM results confirm the formation of AgNPs with an average particle size of 8–12 nm. The TLC, CC and HPLC revealed that four main compounds, pentacosanoic acid (**1**), piperine (**2**), *β*-sitosterol (**3**), and campesterol glucoside (**4**), isolated from *P. chaudocanum* extract act as reducing and stabilizing agents for AgNP formation, and piperine plays a vital role in green synthesis. The chemical structures of these compounds were determined by ESI MS, FTIR, and one- and two-dimensional NMR spectroscopic data analysis. This approach is an efficient, green, cost-effective, eco-friendly and promising technique for synthesizing AgNPs with applications in the colorimetric detection of Hg^2+^ and antibacterial activity.

## 1. Introduction

Silver nanoparticles (AgNPs) possess optical, chemical, electronic and magnetic properties, biocompatibility with low toxicity, leading to various modern applications. However, the chemical reduction-based synthesis of AgNPs typically demands strong reduction such as sodium borohydride, citrate and surfactants, and stabilizing agents, which exposes high risks in operation and produces hazardous byproducts [1]. Alternatives (e.g. alkaloids, flavonoids, terpenoids and phenolic compounds), which are available in microorganisms and plant extracts, have recently been employed in the AgNPs green synthesis [2]. However, in most cases, the mechanism and main compounds responsible for green AgNPs synthesis have not been explored yet. Therefore, the research to find a suitable plant, and determine key compounds for the extract-based green synthesis of AgNPs, is a matter of increasing interest.

The genus *Piper* includes 2000 species, making it one of the largest genera of basal angiosperms. *Piper* is used in condiments, food as well as in medicine worldwide. *Piper* genus contains a variety of compounds, including alkaloids, flavonoids, steroids and terpenoids [3]. Results from the component analysis of the *Piper* genus showed that alkaloids are the most abundant compounds available in the *Piper* genus with about 300 alkaloids in which piperine is an alkaloid, most being isolated [4]. Piperine possesses diverse activities, including anti-inflammatory, anticancer, antiviral, anti-larvicidal, pesticide and antidepressant. In particular, piperine is identified as a bioavailability enhancer, which is a drug facilitator that increases drug biovailability when in a combination [5]. The structure of piperine includes electron-rich components, enabling it to form a complex with metal ions, and act as reductant. Typically, the successful application of piperine and extracts in the *Piper* genus such as *Piper betle*, *Piper chaba*, *Piper nigrum* and *Piper longum* was reported for the AgNPs synthesis [6–8]. Consequently, other species belonging to the *Piper* genus have great potential for the synthesis of AgNPs. *Piper chaudocanum* is a medicinal plant which is widely available and used to treat several diseases such as headache, rheumatism and colds [9]. *P. chaudocanum* also contains bioactive organic molecules that can be used as reductive agents and stabilize colloidal particles in reaction solutions for nanoparticle synthesis processes. For this purpose, the determination of chemical constituents of *P. chaudocanum* and its utilization as an agent for reducing and stabilizing are important issues in the green synthesis strategy of nanomaterials, especially in the synthesis of AgNPs [10].

Mercury (Hg) is a well-known toxic metal that causes serious health and environmental problems. Various sensing methods, including atomic absorption spectrometry, inductively coupled plasma mass spectrometry, and atomic emission spectrometry have been widely used for detecting Hg^2+^ ions. However, these methods require expensive equipment and complex operations, which limit their applications for rapid and on-field analysis. To overcome these drawbacks, the development of biomaterials-based colorimetric sensors has been conducted for Hg^2+^ detection. Recently, the AgNPs-based label-free colorimetric sensors for Hg^2+^ ions in water have attracted more and more attention because of their intrinsically high sensitivity, low limit of detection, and easy colorimetric read-out [11,12]. In these colorimetric assays, the biosynthesized AgNPs are functionalized by piperine, and various other organic compounds containing functional groups such as hydroxy, amine, amide and hetero-aromatic rings that can either stabilize or aggregate AgNPs through electrostatic interactions or hydrogen bonds. AgNPs-based colorimetric sensors for Hg^2+^ ion detection develop from the interaction between the modified surface of nanoparticles and Hg^2+^, inducing a non-crosslinking aggregation or interparticle crosslinking aggregation of nanoparticles, providing characteristic colorimetric responses [11].

Another well-known practical application of AgNPs is as an effective antibacterial agent [13]. This antibacterial property of AgNPs plays an important role in food, medicine, environment and agriculture fields. The antibacterial capability of nanoparticles depends highly on nanoparticles' physico-chemical properties, including stability, size, shape and surface charge, as well as capping agents [14]. These characteristics are believed to be crucial for antibacterial activity. Capping agents such as flavonoids, alkaloids, terpenoids and steroids not only act as the agglomeration of the nanoparticles but also improve the antibacterial action of AgNPs. Therefore, to synthesize AgNPs with an effective antibacterial activity, it is crucial to research plant extracts containing biomolecules that have antibacterial activity of their own. Up to now, there has been no reports on the application of *P. chaudocanum* for AgNPs biosynthesis and their use for antibacterial activity, as well as highly sensitive colorimetric detection of Hg^2+^.

In this study, for the first time, we synthesized AgNPs using the stem extract of *P. chaudocanum*. The biosynthesis factors, including extract amount, temperature and reaction time were thoroughly evaluated. *P. chaudocanum* phytocompounds participating in, and responding to, the green synthesis of AgNPs were identified using column chromatography, spectroscopic methods and HPLC. The synthesized AgNPs were applied to analyze the concentration of Hg^2+^ ions in solution by colorimetric detection, and antibacterial activity. The Hg^2+^ colorimetric detection mechanism was also proposed. The practical application of the designed sensor was further performed using hospital wastewater.

## 2. Material and methods

### 2.1. Chemicals and materials

Piperine (99.5%) and solvents including ethanol (99%), methanol (99.9%), dichloromethane (99.9%), chloroform (99%), acetic acid (99.8%), acetonitrile (99.8%), ethyl acetate (99.5%), *n*-butanol (99.5%), *n*-hexane (99.5%) and silica gel (60G, 0.043–0.063 mm particle size), sephadex LH-20 (99.8%) were collected from Merck chemicals. AgNO_3_ (99.8%), NaOH (99.5%), Hg(NO_3_)_2_ (99.9%) at analytical grade were also purchased from Merck chemicals. Fresh *P. chaudocanum* stems were collected in Son La Province, Vietnam in March 2021, and then washed with deionized water, dried at 50°C, and crushed with a grinder to change into powder form.

### 2.2. Silver nanoparticles synthesis

**Figure 1.**
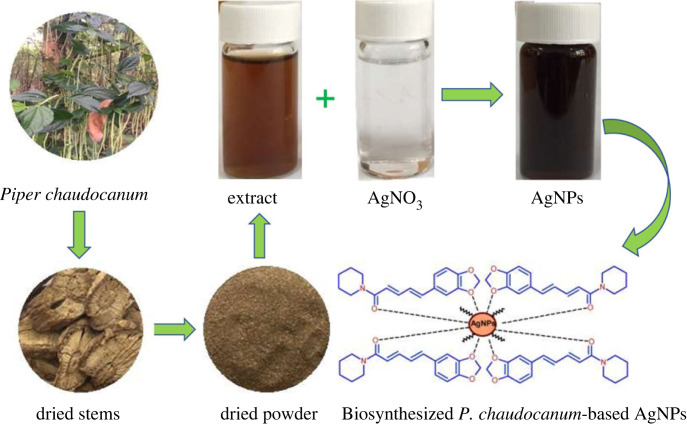
Schematic diagram of AgNPs synthesis using stem extract of *P. chaudocanum.*

Figure 1 illustrates the schematic diagram of AgNPs synthesis using *P. chaudocanum* stem extract. The preparation of the extract of *P. chaudocanum* stems was performed by ultrasonically extracting the dried powder (10 g) in deionized water (100 ml) at 50°C for 1 h, and then slowly added into 10 ml of 1 mM AgNO_3_ solution, under stirring at speed of 800 rpm. The mixture was subsequently added with one drop of 0.1 N NaOH solution and stirred continuously. The AgNPs were obtained by centrifuging at 13 000 rpm for 10 min to remove the impurities and subsequently dispersed into deionized water. Typically, the AgNPs biosynthesis was carried out under various conditions including extract amount (0.25–1.0 ml), reaction temperature (25–80°C), and reaction time (5–40 min) to identify the optimal synthesis conditions in order to achieve a high yield of AgNPs formation with additional consideration of particle size. All samples were measured by UV-Vis spectra using wavelength scanning at the range of 300–800 nm with a spectrophotometer (UH5300, Hitachi, Japan), and at scan speeds of 400 nm/min. Characterizations of AgNPs synthesized at the optimal conditions were analysed using various techniques, including X-ray diffractometer (D2-Phaser, Brucker, Japan) at 2*θ* of 20–80° equipped with a Cu K*_α_* tube and a Ni filter (*λ* = 0.1542 nm) with 0.05° per step, FT-IR spectra (Perkin Elmer Spectrum Two) at a wavenumber band of 4000–400 cm^−1^, and scanning electron microscopy (SEM-Hitachi SU8000) operated in the high mag mode 250 K at an acceleration voltage of 5 kV, and high-resolution transmission electron microscopy (TEM-JEOL 2100F) operated at 80 kV, direct mag 100 K.

### 2.3. Chemical constituents of *Piper chaudocanum* stems

#### 2.3.1. Qualitative phytochemical tests of plant extract

To detect the availability of plant constituents (e.g. alkaloids, flavonoids, steroids, terpenoids, tannins and phenolic compounds) in the stem ethanol and water extracts of *P. chaudocanum*, the preliminary phytochemical screening was done by standard methods such as alkaloids using Mayer's, Wagner's and Dragendorffs’ reagent; flavonoids with lead acetate test, and zinc hydrochloric acid reduction test; steroids with Salkowski's test, and terpenoids with copper acetate test, and Noller's test; and tannins and phenolic compounds with ferric chloride test. They were identified using standard procedures by characteristic colour changes and precipitation reactions [15,16]. The ethanol and aqueous *P. chaudocanum* extracts were obtained by ultrasonically extracting dried powder in ethanol and distilled water, respectively, and then evaporated in vacuum.

#### 2.3.2. Isolation and identification of *P. chaudocanum* compounds

**Figure 2.**
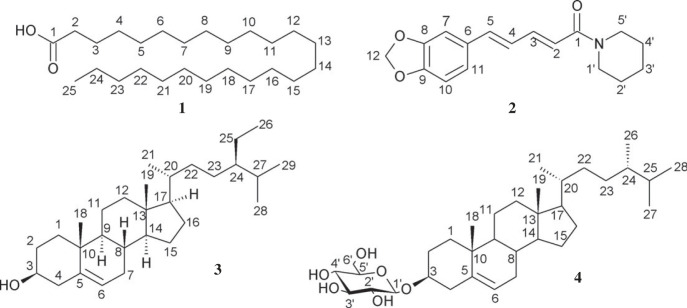
Chemical structures of 1–4 isolated from *P. chaudocanum* stems.

To identify the key compounds in *P. chaudocanum* stem responsible for the formation of AgNPs, the isolation and structural analysis of compounds were carried out. The dried powder (2.0 kg) of *P. chaudocanum* stems was sonicated with 95% ethanol (three times, each 3 L) at ambient temperature. After solvent separation, the EtOH extract (250 g) was suspended with water, then partitioned with EtOAc and *n*-BuOH to gain ethylacetate extract (PCE, 85 g), and butanol extract (PCB, 58 g). The PCE (85 g) was subjected to silica gel column chromatography (CC), eluting with gradient *n*-hexane: EtOAc (100:0–0:100, v/v) to furnish 10 fractions (E1 to E10). Fraction E3 was separated by silica gel CC with the use of a solvent system of *n*-hexane: CH_2_Cl_2_ (95:5, v/v) as eluent to give compound **1** (25 mg). Fraction E5 was chromatographed on a silica gel CC eluting with *n-*hexane: EtOAc (8:2, v/v) to gain five smaller sub-fractions, E5.1 – E5.5. E5.2 (200 mg) was re-crystallized with chloroform to get a pure compound **2** (40 mg). The separation of the PCB (58 g) was conducted using CC on silica gel, stepwise gradient elution with CH_2_Cl_2_:−MeOH to afford seven fractions (B1 to B7). Fraction B3 (1.2 g) was further purified using a Sephadex LH-20 CC column, eluting with CH_2_Cl_2_:−MeOH (1:9, v/v) to obtain compound **3** (15 mg). Compound **4** (20 mg) was isolated from fraction B4 (432 mg) utilizing a two-stage separation, commencing with silica gel CC eluted with CH_2_Cl_2_:−MeOH (4:1, v/v), thereafter by silica gel CC eluted with CH_2_Cl_2_:−MeOH:−H_2_O (4:1:0.1, v/v). The extraction and isolation of compounds **1–4** from *P. chaudocanum* stems was described in scheme 1 of electronic supplementary material. The compounds' chemical structures (figure 2) were characterized by FT-IR, UV-Vis, MS, one- and two-dimensional NMR analysis and compared with reported data.

Thin-layer chromatography (TLC) was performed on pre-coated plates (silica gel 60 F_254_), compounds were visible with a UV lamp at 365, and 254 nm wavelength and spots were detected by spraying with vanillin-sulfuric acid. CC was conducted using silica gel (60G, 0.043–0.063 mm particle size (Merck)), and chromatograhpy columns (10–30 mm × 100–500 mm, Schott Duran Germany). FT-IR analysis was reported on Perkin Elmer Spectrum Two. UV-visible spectra was recorded on UH5300, Hitachi, Japan. NMR spectra were measured by a Bruker Advance I (500 MHz), CDCl_3_, and MeOD were solvent used for NMR analyses. Chemical shifts (*δ*) are provided in parts per million (ppm) and coupling constants (*J*) in hertz (Hz). MS was recorded on LC-MSD-Trap-SL, Varian instrument, USA.

#### 2.3.3. Content analysis of the extract

100 mg powders of *P. chaudocanum* stems were dissolved with 10 ml of methanol. A 10 mg ml^−1^ sample solution was produced by sonicating the prepared sample within 10 min, and then filtering (0.22 µm). The sample solution obtained was subjected to the HPLC experiment.

For determining the crucial compounds presented in *P. chaudocanum* extract, HPLC analysis was performed. Stock solutions (5 mg ml^−1^) of piperine were produced by dissolving 250 mg of piperine in 50 ml of methanol. The six different concentrations of 0.125, 0.5, 0.75, 1.0, 1.25 and 1.5 mg ml^−1^ were prepared by diluting from stock solution. The sample solution above with 10 mg ml^−1^ concentration was used for HPLC analysis. The method conditions were the mobile phase composition of acetonitrile: water: acetic acid (60:39.5:0.5, v/v), at 1 ml/min of flow rate, and 10 ml of injection volume. By comparing the substances to the reference compounds and utilizing the retention duration, and the absorption spectrum profile, investigated compounds were identified by chromatographic analysis using the HPLC. The chemical substances were analysed by using a Waters Arc HPLC system with a PDA detector (200**–**800 nm), and a silica gel C_18_ column (5 µM particle size, 25 cm × 4.6 mm).

### 2.4. Determination of antibacterial activity of AgNPs by zone inhibition assay

Biosynthesized AgNPs' antibacterial activity was tested against Gram-negative bacteria (*Escherichia coli* from ATCC 25922 and *Pseudomonas aeruginosa* from ATCC 15442) and Gram-positive bacteria (*Staphylococcus aureus* from 25923) by the agar disc diffusion method [17]. Firstly, 100 µL bacterial suspensions with a concentration of 4–5 × 10^8^ CFU/ml were spread on Luria-Bertani (LB) agar plates. Five wells (diameter of 6 mm) were drilled in each of these plates with a sterile borer. Then, 50 µl of AgNPs solution at three different concentrations (25, 50 and 100 µg ml^−1^) and extracts at the concentration of 100 µg ml^−1^ were placed into each well, and 2% DMSO solution as a negative control was poured into separated wells. Lastly, the inhibition zones on the tested plates were measured after a 24-h incubation period at 37 ± 2°C.

### 2.5. Application of synthesized AgNPs as a colorimetric sensor for Hg^2+^ detection

#### 2.5.1. Detection of Hg^2+^ ions

To evaluate Hg^2+^ detection capacity of synthesized AgNPs as a colorimetric sensor, the sensitivity, the minimal Hg^2+^ detectable concentration in aqueous solution, and the detecting probe's reaction time were observed from the changes in the solution's colour and measuring the UV-Vis absorption. The various concentrations (0.05 µM to 10.0 µM) of Hg^2+^ (0.5 ml) were added to the AgNPs solution (3 ml) which was diluted three times with deionized water. The limit of detection (LOD) of the Hg^2+^ colorimetric detection by the synthesized AgNPs was calculated based on following equation: [18]

2.1 $$\mathrm{LOD}=\frac{3xS_{y}}{b}$$

where *S*_y_: standard deviation of response (peak area); *b*: slope of calibration curve.

To identify the stability of AgNPs, the optimized solution of nanoparticles was kept in the dark and measured by UV-Vis spectra after two months. To determine the response time for the Hg^2+^ detection of biosynthesized AgNPs, the UV-Vis spectroscopy of mixtures after the addition of Hg^2+^ (1 µM) into AgNPs solution was carried out at different detection times.

#### 2.5.2. Analysis of real sample

Evaluation of the proposed method for Hg^2+^ ions determination was performed in real wastewater samples using the standard addition procedure. The wastewater sample was collected from Thai Nguyen hospital and filtered. Different concentrations of Hg^2+^ were spiked into 5 ml of the solution containing hospital wastewater (2 ml), and AgNPs (3 ml). UV-Vis spectra measurement of each sample was conducted in triplicate. To evaluate the relative accuracy of the analysis, it is necessary to carry out the repeatability and stability. The real sample was analysed five times for the repeatability.

#### 2.5.3. Data analysis

Mean values, standard deviation (s.d.) and relative standard deviation (RSD) were calculated using Excel (Microsoft Office 2019). To find the factors with significant effects on AgNPs synthesis and the optimum conditions, statistical analysis including One-way ANOVA and means comparisons was performed using Origin Pro 2021.

## 3. Results and discussion

### 3.1. AgNPs synthesis

**Figure 3.**
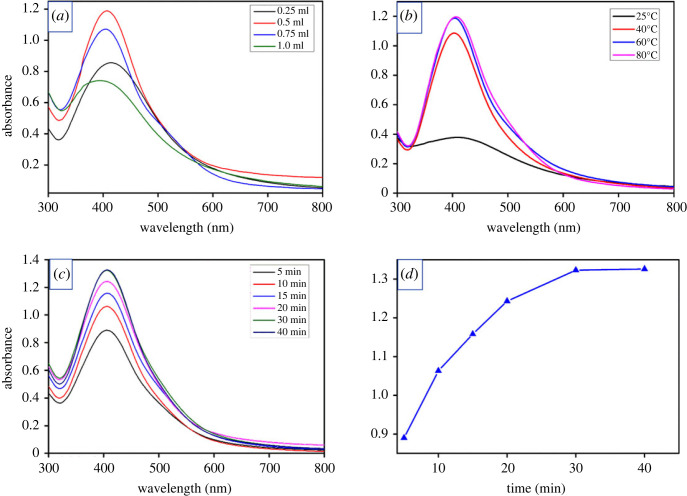
UV-Vis absorption spectra of AgNPs at (*a*) different amounts of extract, (*b*) different temperatures, (*c*) different times and (*d*) absorption intensity versus reaction time.

AgNPs formation could be visually observed by a colour change from pale brown to dark brown. As can be seen from figure 3*a*, the absorption intensity increased significantly as the amount of extract increased from 0.25 to 0.5 ml and all Ag^+^ ions were reduced and stabilized almost completely with 0.5 ml of the extract. However, further increasing the amount from 0.75 ml to 1.0 ml resulted in a decrease in absorption intensity. This could be due to the excess extract left in the solution after the complete formation of AgNPs. Besides, the slightly blue shift of the SPR from 414 to 396 nm when increasing the extract volume indicated decreases in the size of AgNPs. However, at extract volume of 1.0 ml, the broadening of the peak was observed. It can create an increase in the aggregation of nanoparticles. In addition, as the reaction time and temperature increased to 30 min and 60°C, the formation of AgNPs increased as shown in figure 3*b–d*. By further increasing the reaction time and temperature, the absorption intensity remained almost unchanged. This means that the production of AgNPs using *P. chaudocanum* extract took only 30 min to complete, which was significantly less time than the previous report [11]. In addition, we further conducted one-way ANOVA and comparisons between means of absorption intensities to find whether these factors statistically affected the responses (i.e. absorption intensities) and which means were statistically different. As can be seen from electronic supplementary material, table S1, S2 and S3, the changes of extract amounts, reaction temperature and time significantly affected the absorption intensities of AgNPs (*p* ≤ 0.05). The detailed means comparisons additionally confirmed that the absorption intensities from different extract amounts were significantly different (electronic supplementary material, figures S1–S3 and electronic supplementary material, table S1–S3). Similarly, the adsorption intensities from different temperatures as well as times were also significantly different except the means at the temperatures of 60 and 80°C and the means at the times of 30 and 40 min. Hence, the optimal conditions for synthesis of AgNPs were selected at 10 ml of 1 mM AgNO_3_ solution, 0.5 ml of the extract volume, at 60°C in 30 min. Moreover, the *P. chaudocanum* extract can be stored for a long period at room temperature without breakdown, which possibly provides the synthesized AgNPs with high repeatability and stability over time.

### 3.2. Characteristics of AgNPs

**Figure 4.**
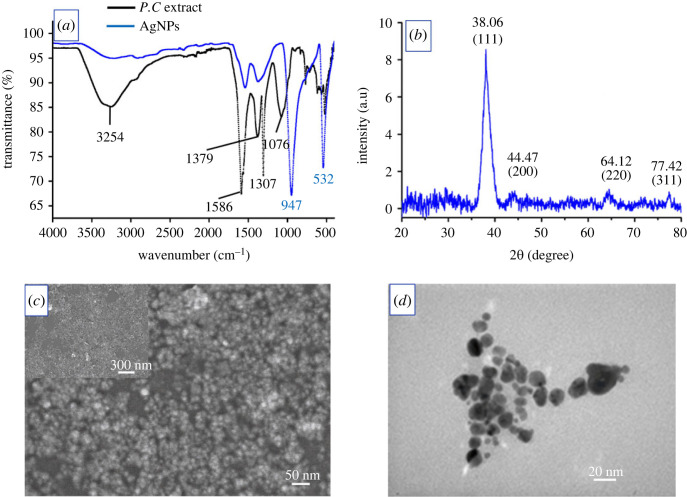
(*a*) FT-IR spectra of the extract and AgNPs, (*b*) XRD pattern, (*c*) SEM image and (*d*) TEM image.

The properties of AgNPs synthesized at the optimal conditions were characterized by FT-IR, XRD, SEM and TEM analyses. The FT-IR spectrum of *P. chaudocanum* stem extract (figure 4*a*) had a characteristic absorption broad band of stretching vibration of the OH group at 3254 cm^−1^. Other absorption bands that appeared at 1586 and 1076 cm^−1^ are C = C aromatic ring stretching or asymmetric C = O stretching vibrations, and C-O stretching vibrations, respectively. Further, the strong band at 1379 and 1307 cm^−1^ could be attributed to the C-N stretching vibration of N-C = O moiety in piperine [19]. The presence of these groups in the FT-IR spectra indicates that the plant extract contained significant components that act as reducing agents. In comparison, the peaks in the FT-IR spectra of AgNPs were shifted and decreased in intensity due to the molecules of the extract having the affinity to interact with AgNPs, resulting in the molecules acting as stabilizers of AgNPs. Moreover, the appearance of characteristic absorption of a peak with wavenumbers of 947 and 532 cm^−1^ may correspond to the N-H out-of-plane bending vibration, and the vibration of Ag-O ionic bonds. In addition, the EDX spectrum (electronic supplementary material, figure S4) shows elemental signals of silver atoms and other elements such as nitrogen, chlorine and carbon, which may be attributed to the bioactive molecules that are present in the *P*. *chaudocanum* stem extracts. In the XRD pattern of AgNPs (figure 4*b*), the Bragg's reflection was observed at 2*θ* values of 38.06, 44.47, 64.12 and 77.42^o^, representing the planes of (111), (200), (220), and (311) which corresponded to the face-centered cubic crystal structure of AgNPs [20]. The SEM and TEM images reveal that the AgNPs had been mostly amalgamated into nanoclusters (figure 4*c,d*). The nanoparticles appeared generally in irregular shape with an average size range of 8–12 nm. The size distribution of synthesized AgNPs (electronic supplementary material, figure S5) was measured using Image J Software. Biosynthesized AgNPs were uniformly distributed.

### 3.3. Chemical constituents of *P. chaudocanum*

**Figure 5.**
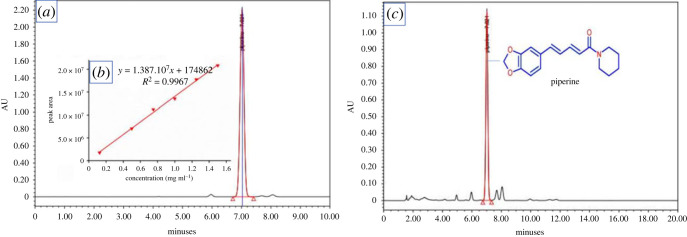
(*a*) HPLC chromatogram of standard *piperine,* (*b*) calibration curve of piperine and (*c*) HPLC chromatogram of *P. chaudocanum* extract.

To identify the *P. chaudocanum* phytocompounds participating and responding to the green synthesis of AgNPs*,* the qualitative phytochemical test, isolation and identification of components and HPLC analysis was performed. Alkaloids, flavonoids, triterpenoids, steroids, tannins and phenolic compounds were found to be available in the ethanol and aqueous extracts of *P. chaudocanum* stems (electronic supplementary material, table S4). These compositions are also present in most of extracts of other species in *Piper* genus [3]. After checking by thin-layer chromatography (TLC) each fraction of the EtOAc, and *n*-BuOH extracts of *P. chaudocanum* stem, and using various chromatography methods, four main compounds including a fatty acid, pentacosanoic acid (**1**) [21], an amide alkaloid, piperine (**2**) [22,23], and two steroids, *β*-sitosterol (**3**) [24] and campesterol glucoside (**4**) [25,26], were isolated. The NMR spectral data of compounds **1–4** were consistent with those previously reported in the literature (electronic supplementary material, figures S6–S20). Compounds **2–4** also were isolated from many species in *Piper* genus, and compound **1** was isolated for the first time from this *Piper*. As a result, these compounds can be argued to play both key roles in the extract-mediated green synthesis of AgNPs. Compounds **2–3** exhibit a high antibacterial activity against Gram-negative and Gram-positive bacteria [5,27]. Moreover, the HPLC analysis results (figure 5) indicate that piperine at 0.736 mg ml^−1^ was a major compound of *P. chaudocanum*, and responsible for transferring the charge in the Ag^+^ reduction to AgNPs. This result is compatible with the result of TLC analysis of EtOAc, and *n*-BuOH extracts. As a result, piperine plays a crucial role in green synthesis.

**Figure 6.**
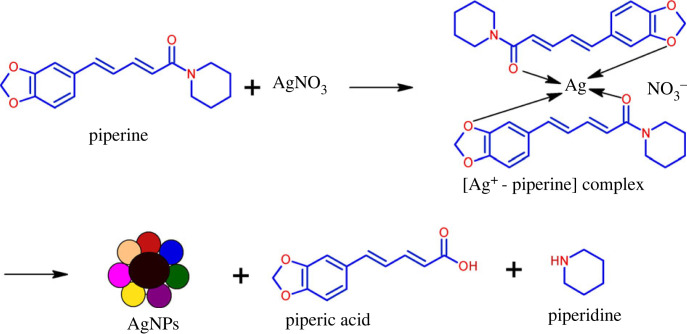
Proposed mechanism of *P. chaudocanum* stem extract-mediated synthesis of AgNPs.

The proposed mechanism of *P. chaudocanum* stem extract-mediated synthesis of AgNPs with piperine as a major reducing agent is shown in figure 6. Firstly, Ag^+^ will bind to the amide group of piperine to form an intermediate complex of Ag^+^ with piperine. Subsequently, through reduction phenomena, AgNPs are formed, while piperine is converted into piperic acid and piperidine. Consequently, the formed AgNPs are stabilized by organic compounds such as pentacosanoic acid, piperine, *β*-sitosterol and campesterol glucoside together with other components.

### 3.4. Antibacterial activity of AgNPs

**Figure 7.**
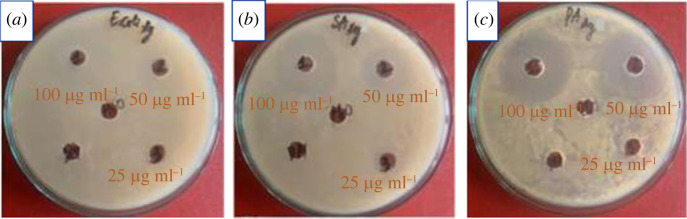
Antibacterial activity of AgNPs against: (*a*) *E. coli*, (*b*) *S. aureus*, (*c*) *P. aeruginosa.*

In this experiment, we further tested the antibacterial ability of the biosynthesized AgNPs against pathogenic bacteria using the agar well diffusion method on three strains including *E. coli*, *P. aeruginosa* (gram-negative) and *S. aureus* (gram-positive). As a result, AgNPs showed an effective inhibition toward all the tested strains (figure 7). The diameters of zones were displayed in electronic supplementary material, table S5. It is clear that the diameters of inhibition zones for all bacteria were raised with increasing concentrations of AgNPs. The growth inhibition activity of AgNPs at 100 µg/ml concentration against *P. aeruginosa*, *S. aureus* and *E. coli* was obtained to be 28 ± 0.1 mm, 22 ± 0.1 mm, and 22 ± 0.2 mm, respectively. These results may be explained owing to the small size of the nanoparticles, and biomolecules such as piperine, *β*-sitosterol and campesterol glucoside attached over nanoparticle surfaces for their antibacterial action. According to a recent study, nanoparticles with a size of 10–15 nm have higher antibacterial action because they can adhere to membranes and subsequently enter bacteria [13]. Hence, *Piper chaudocanum* stem extract-mediated AgNPs with an average particle size of 8–12 nm exhibited strong antibacterial activity. Additionally, *P. chaudocanum* extracts (electronic supplementary material, table S6 and electronic supplementary material, figure S21), especially compounds **2–3** also exhibited a strong inhibition efficiency toward all the above bacterial strains, and as a result, a synergistic effect of synthesized AgNPs, and the capping components improve the antibacterial action. Therefore, the synthesized *P. chaudocanum* extract mediated AgNPs may be used as a powerful antibacterial agent in bio-related applications.

### 3.5. Hg^2+^ detection capacity of synthesized AgNPs

**Figure 8.**
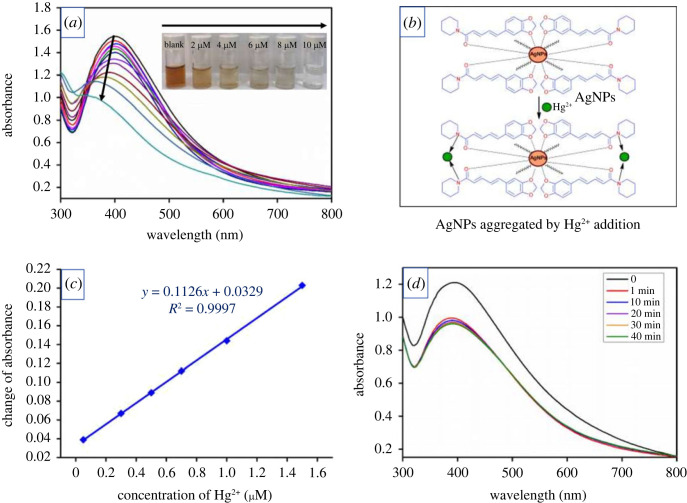
(*a*) UV-Vis absorbance spectra of mixture solutions between AgNPs and Hg^2+^ with various Hg^2+^ concentrations (0–10 µM); insert: picture of colour changes of mixture solutions as a function of Hg^2+^ concentration (bottles from left to right corresponding to Hg^2+^ concentrations from 0 to 10 µM); (*b*) mechanism scheme for the Hg^2+^ induced colorimetric sensing response of AgNPs; (*c*) regression co-efficient plot between the concentration of Hg^2+^ versus absorbance; and (*d*) time-dependent UV-Vis spectra of AgNPs solution in the presence of Hg^2+^ ions.

The synthesized AgNPs may be suggested as a good colorimetric probe for Hg^2+^ ion detection because of the interaction between Hg^2+^ ions and free functional groups of the molecules on the surface of AgNPs. As can be seen from figure 8*a*, the surface plasmon resonance (SPR) peak intensity reduced gradually and the shifts toward lower wavelengths were observed on the increase of Hg^2+^ concentration from 0.05 to 10.0 µM, corresponding to the colour changes of solutions from dark brown to colourless (inset of figure 8*a*). The mechanism of Hg^2+^ colorimetric detection is proposed in figure 8*b*. The selective detection of Hg^2+^ would be due to the following possible mechanisms: (i) interactions between AgNPs and Hg^2+^ through N–Hg^2+^–N linkages, by assisting the reduction of Hg^2*+*^ to Hg and oxidation of AgNPs to Ag^+^; and (ii) aggregation of AgNPs, resulting in the colour change. The photograph in figure 8*a* also presents the colour changes of mixture solutions as a function of Hg^2+^ ions concentration, which could be seen with the naked eye starting at 4 µM of Hg^2+^ ions*.* From figure 8*c*, there was a linear relationship between the change of absorbance and Hg^2 +^ concentration over the range of 0.05–1.5 µM at 400 nm (*y* = 0.1126*x* + 0.0329, *R*^2^ = 0.9997). Based on equation (2.1), the LOD for colorimetric Hg^2+^ detection by the synthesized AgNPs was calculated to be 23 nM. We then compared LOD from our detection method to those from reported data (table 1). Obviously, table 1 shows that the LOD value reported in this study is below the allowed maximum level of drinking water from WHO (i.e. 30 nM) and comparable with LOD in the previous publications (table 1). Especially, the SPR peak intensity immediately decreased after adding Hg^2+^ (1 µM) to AgNPs solution. AgNPs synthesized using the extract of *P. chaudocanum* stems were highly stable as shown in electronic supplementary material, figure S22. UV-Vis spectra of AgNPs has not changed in SPR band, shape and broadening of peak width after two months. The intensity decreased insignificantly from 1.519 to 1.499 at 400 nm. Moreover, no significant change was observed after 1 min (figure 8*d*)*.* This suggested the potential application of the biosynthesized AgNPs for rapidly visual colorimetric detection of Hg^2+^ ions with high sensitivity and low limit of detection level without any modification.

**Table 1.** Comparison of LOD using *P. chaudocanum* stem - AgNPs with other NP-based colorimetric methods for Hg^2 +^ determination.

metal nanoparticles	detection method	LOD	ref.
the sodium salt of N-cholyl-*L*-cysteine-AgNPs	colorimetric	8 nM	[28]
graphene oxide – AgNPs	electrochemical	0.285 µM	[29]
lotus root nitrogen-doped carbon dots	fluorescence	18.7 nM	[30]
*Cinnamomum tamala* extract – AuNPs	colorimetric	0.49 µM	[31]
*Ziziphus mauritiana* leaf – AgNPs	colorimetric	0.04 nM	[12]
*garlic extract* – AgNPs	colorimetric	15.7 nM	[32]
*Cochlospermum gossypium bark* – AgNPs	colorimetric	50 nM	[33]
*Agaricus Bispores*-AgNPs	electrochemical	2.1 µM	[34]
*P. chaudocanum* stem – AgNPs	colorimetric	23 nM	present study

### 3.6. Determination of Hg^2+^ ion water samples

The practical application of the designed sensor was further evaluated using the hospital wastewater. The recoveries were in the range of 94.1***–***103.3% (table 2) with the relative standard deviation (RSD) less than 5% suggesting that our sensing system could be suitable for practically analysing Hg^2+^ in real wastewater samples. The UV-Vis spectra corresponding to the real water samples was shown in electronic supplementary material, figure S23.

**Table 2.** Hg^2+^ determination in hospital wastewater using the synthesized AgNPs.

sample	spiked concentration (µM) (mean ± s.d.)	found concentration (*n* = 5) (µM) (mean ± s.d.)	recovery (%)	RSD (%)
hospital wastewater	0	0	100	0
	0.30 ± 0.01	0.31 ± 0.01	103.3	3.22
	0.51 ± 0.04	0.48 ± 0.02	94.1	4.17
	1.00 ± 0.03	1.01 ± 0.05	101.0	4.95

± s.d.: standard deviation; RSD: relative standard deviation.

## 4. Conclusion

In summary, this study reports for the first time the successful synthesis of AgNPs using the stem extract of *P. chaudocanum* which was then effectively employed for Hg^2+^ colorimetric detection in a solution. The results of XRD, SEM and TEM analyses show that the synthesized AgNPs have a face-centred cubic crystal structure and an average size of about 8–12 nm. The optimal conditions for the synthesis of AgNPs were using 0.5 ml of the extract with a reaction time of 30 min at 60°C. A fatty acid, pentacosanoic acid (**1**), an amide alkaloid piperine (**2**), and two steroids, *β*-sitosterol (**3**) and campesterol glucoside (**4**), were isolated from *Piper chaudocanum* extract to act as reducing and stabilizing agents for AgNPs formation, and piperine plays a key role in green synthesis. The colorimetric responses can be observed by the naked eye and measured by UV-Visible spectrometry without using a complicated pretreatment procedure. This colorimetric detection provides a simple, rapid, and low-cost route with an LOD of 23 nM for the measurement of Hg^2+^ ions in an aqueous solution, as well as in real wastewater. The biosynthesized AgNPs also show effective antibacterial activity against all the tested bacterial strains.

## Data Availability

All data relevant to this work are deposited at the Dryad Digital Repository: https://doi.org/10.5061/dryad.tht76hf23 [35]. The data are provided in electronic supplementary material [36].
